# Monitoring of Target-Site Mutations Conferring Insecticide Resistance in *Spodoptera frugiperda*

**DOI:** 10.3390/insects11080545

**Published:** 2020-08-18

**Authors:** Debora Boaventura, Macarena Martin, Alberto Pozzebon, David Mota-Sanchez, Ralf Nauen

**Affiliations:** 1 Institute of Crop Science and Resource Conservation, University of Bonn, 53115 Bonn, Germany; de.boaventura@hotmail.com; 2 Bayer AG, Crop Science Division, R&D Pest Control, 40789 Monheim, Germany; 3 Department of Agronomy, Food, Natural Resources, Animals and Environment, University of Padova, 35020 Padova, Italy; macamar_20@hotmail.com (M.M.); alberto.pozzebon@unipd.it (A.P.); 4 Department of Entomology, Michigan State University, East Lansing, MI 48824, USA; motasanc@msu.edu

**Keywords:** fall armyworm, insecticide resistance, target-site mutations, Bt resistance, corn strain, rice strain, resistance management, Indonesia, Kenya

## Abstract

**Simple Summary:**

Fall armyworm, *Spodoptera frugiperda*, is an invasive moth species and one of the most destructive pests of maize. It is native to the Americas but recently invaded (sub)tropical regions in Africa, Asia and Oceania. Fall armyworm larvae feeding on maize plants cause substantial economic damage and are usually controlled by the application of insecticides and genetically modified (GM) maize expressing *Bacillus thuringiensis* (Bt) proteins, selectively targeting fall armyworm. It has developed resistance to many different classes of insecticides and Bt proteins as well; therefore, it is important to check field populations for the presence of mutations in target proteins conferring resistance. Here, we developed molecular diagnostic tools allowing us to test the frequency of resistance alleles in field-collected populations, either alive or preserved in alcohol. We tested 34 different populations collected on four different continents for the presence of mutations conferring resistance to common classes of insecticides and Bt proteins. We detected resistance mutations which are quite widespread, whereas others are restricted to certain geographies or even completely absent. The established molecular methods show robust results in samples collected across a broad geographical range and can be used to support decisions for sustainable fall armyworm control and applied resistance management.

**Abstract:**

Fall armyworm (FAW), *Spodoptera frugiperda*, a major pest of corn and native to the Americas, recently invaded (sub)tropical regions worldwide. The intensive use of insecticides and the high adoption of crops expressing *Bacillus thuringiensis* (Bt) proteins has led to many cases of resistance. Target-site mutations are among the main mechanisms of resistance and monitoring their frequency is of great value for insecticide resistance management. Pyrosequencing and PCR-based allelic discrimination assays were developed and used to genotype target-site resistance alleles in 34 FAW populations from different continents. The diagnostic methods revealed a high frequency of mutations in acetylcholinesterase, conferring resistance to organophosphates and carbamates. In voltage-gated sodium channels targeted by pyrethroids, only one population from Indonesia showed a mutation. No mutations were detected in the ryanodine receptor, suggesting susceptibility to diamides. Indels in the ATP-binding cassette transporter C2 associated with Bt-resistance were observed in samples collected in Puerto Rico and Brazil. Additionally, we analyzed all samples for the presence of markers associated with two sympatric FAW host plant strains. The molecular methods established show robust results in FAW samples collected across a broad geographical range and can be used to support decisions for sustainable FAW control and applied resistance management.

## 1. Introduction

The fall armyworm (FAW), *Spodoptera frugiperda* (J.E. Smith) (Lepidoptera: Noctuidae), is an important agricultural pest of several crops in the western hemisphere [1,2]. Since 2016, FAW distribution expanded globally by invading different continents, first reported in Africa and later reaching Southeast Asia and, more recently, Australia, totalizing its presence in 107 countries worldwide [3,4,5,6]. The success of FAW spread is due to many factors, such as the high reproductive capacity, long-distance migration and high polyphagia [7,8].

Two sympatric host plant strains of *S. frugiperda* have been previously described: the corn strain, which feeds on large grasses such as corn and sorghum, and the rice strain, preferring small grasses such as rice [9,10]. The two strains differ not only in their host preferences but also regarding their physiology [11], insecticide susceptibility [12] and composition of genes involved in chemoreception, detoxification and digestion [13].

In South America, both strains have been already identified in field populations using molecular markers, and most populations were structured in agreement with their host preferences [14,15]. Although initial studies on the genetic structure of FAW populations from the newly invaded countries suggest a common source of origin, probably from Florida or the Caribbean [16,17], there are differences in the strain haplotypes and disagreements regarding the molecular marker and host plant that may imply inter-population movement of FAW populations from African and Asian countries [18].

An understanding of the genetic background of FAW is essential for resistance management strategies in different regions. Besides the intrinsic variation in insecticide susceptibility associated with FAW strains [11,12,19,20], the impact of migration on insecticide resistance will depend on the pre-existence of resistance alleles in the starting population and selection pressure on the newly invaded areas and spread [14,17].

At present, the Arthropod Pesticide Resistance Database (APRD) reports 144 cases of insecticide resistance in FAW globally. Among the 41 different active substances affected, 45% of the cases belong to proteins produced by *Bacillus thuringiensis* (Bt), 26% and 19% to insecticides targeting the voltage-gated sodium channel (VGSC), and acetylcholinesterase (AChE), respectively [21]. The high number of cases reported for Bt proteins, particularly those expressed in transgenic corn, reflects the intensive adoption of transgenic crops, which corresponded to 191.7 million ha worldwide in 2018 [22]. The adoption of transgenic crops expressing insect-resistant traits to control lepidopteran pests is most advanced in the United States and Brazil. Nevertheless, in Asia, the adoption of Bt-corn is high, particularly in China and India, while it is rather limited to just a few countries in Africa [22].

Many resistance cases are reported for pyrethroid insecticides targeting the VGSC and inhibitors of AChE (i.e., carbamates and organophosphates). This is due to low application costs, a high number of compounds registered for decades and frequent applications [23]. Nevertheless, together, they still account for around 30% of the global insecticide market share [23]. The most modern chemical class used to control lepidopteran pests are the diamide insecticides, acting on the ryanodine receptor (RyR) and used in different agronomic settings [23].

It is unclear whether FAW populations present in Africa were already resistant to old chemical compounds [24]. However, farmers have complained about the efficacy of pyrethroids and organophosphate insecticides under field conditions [25]. Hence, this has led to misuse by increasing rates, application frequency or even the use of unregistered compounds [25,26]. On the other hand, if no control measures against FAW are adopted, the yield losses for corn could reach up to 20.6 m tons per annum for only 12 corn-producing countries in Africa [24].

Insecticide resistance is usually conferred by the insensitivity of the target receptor and/or pharmacokinetic processes modifying the rate or the properties of the insecticides delivered to the target site [27]. Amino acid substitutions/indels at the VGSC (T929I, L932F, and L1014F), AChE (A201S, G227A, and F290V), RyR (I4790M and G4946E) and ATP-binding cassette subfamily C2 transporter (ABCC2) (GC insertion and GY deletion) have been linked to resistance in *S. frugiperda* to pyrethroids, carbamates and organophosphates, diamides and Bt proteins (e.g., Cry1F), respectively [28,29,30].

In the present study, we monitored the frequency of the above-mentioned target-site mutations in the VGSC, AChE, RyR and ABCC2 in 34 populations of *S. frugiperda* collected in Brazil, Puerto Rico, Kenya and Indonesia by PCR-based allelic discrimination assays as well as pyrosequencing diagnostics. We validated and established robust diagnostic tools based on genomic DNA, which can be implemented to support decisions for appropriate resistance management strategies.

## 2. Materials and Methods

### 2.1. Insect Collection

**Figure 1:**
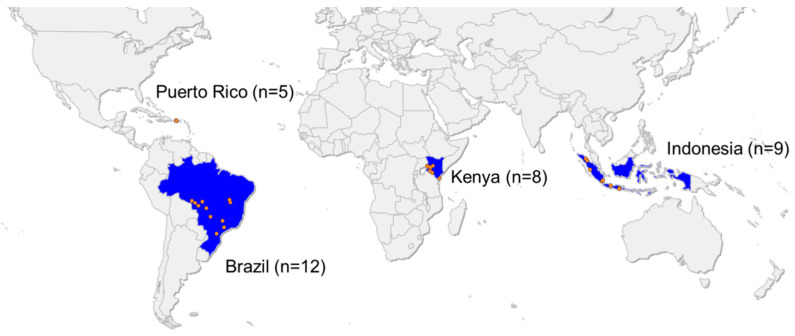
Map showing the origin of 34 fall armyworm populations collected in Brazil, Puerto Rico, Kenya and Indonesia (more details about collection sites in Table S1). All samples were used for the genotyping of target-site mutations. The schematic map was created using EasyMap software (Lutum + Tappert DVBeratung GmbH, Bonn, Germany).

Larvae of FAW were collected from different sites in Brazil, Puerto Rico, Kenya and Indonesia (Figure 1 and Table S1) and kept in 70% ethanol or RNAlater^®^ (Life Technology, Carlsbad, CA, USA) until DNA extraction and genotyping.

### 2.2. DNA Extraction

Genomic DNA was extracted from individual larvae (whole body for second/third instar and abdominal fragments of fifth instar larvae). At least five individuals per FAW population (Table S1) were used for gDNA extraction and the total sample number used for the genotyping analysis is shown in Table 1. Agencourt DNAdvance™ (Beckmann Coulter, Beverly, CA, USA) and DNeasy Blood & Tissue Kit (QIAGEN, Hilden, Germany) were used to extract gDNA for the pyrosequencing and fluorescent probe assay, respectively. Both kits were used according to the suppliers’ recommended protocols.

### 2.3. PCR and qPCR Conditions

PCR for pyrosequencing, PCR-RFLP and PCR for sequencing were performed in 30 μL reaction mixture containing 15 μL JumpStart™ Taq ReadyMix™ (Sigma-Aldrich, St. Louis, MO, USA), 500 nM of forward and reverse primers (Table S2), around 20 to 50 ng gDNA and nuclease-free water. The cycling conditions comprised 95 °C for 3 min, followed by 40 cycles of 95 °C for 30 s, the respective annealing temperature according to Table S2 for 30 s and 72 °C for 45 s, and a final elongation step at 72 °C for 5 min.

The fluorescent probe assays for detection of mutations F290V, I4790M and a GC insertion in the ABCC2 consisted of reactions set up at a final volume of 10 µL, with 5 µL SsoAdvanced™ Universal Probes Supermix (Bio-Rad, Hercules, CA, USA), 700 nM of forward and reverse primers (Table S2), 200 nM of probes, 20–50 ng of gDNA and nuclease-free water, and the reactions were run in duplicate. The conditions of PCR amplification were 95 °C for 5 min and 40 cycles at 95 °C for 15 s and 60 °C for 30 s. The real-time PCR was conducted in a CFX-384 real-time thermocycler (Bio-Rad, Hercules, CA, USA) and the end-point fluorescence values, taking cycle 35 as a threshold, were plotted in a scatter-plot using Bio-Rad qPCR analysis software CFX Maestro 1.0 (Bio-Rad, Hercules, CA, USA).

### 2.4. Characterization of S. frugiperda Strains

#### 2.4.1. Characterization of COI Haplotypes Using PCR-RFLP

Corn and rice strain genotyping were performed using the molecular markers based on mitochondrial cytochrome oxidase subunit I (COI) with polymerase chain reaction-restriction fragment length polymorphism (PCR-RFLP), according to Nagoshi et al. (2007, 2012) [31,32]. Three to five individuals from different populations of *S. frugiperda* collected in Kenya (EP-K, KV-K, NJ-K, MJ-K and MD-K), Indonesia (WS-I, DS-I, S-I, WC-I and BC-I), Brazil (Sf_Bra, Sf_Cor, MT-PL1-2, BA-SD and PR-PG) and Puerto Rico (PR60, PR61, PR62, PR63 and PR64) (Table S1) were characterized. PCR reactions were carried out according to Section 2.3, using primer JM76 and JM77 (Table S2). After amplification, 1.0 μL of FastDigest MspI (Thermo Scientific, Vilnius, Lithuania) was added to 10 μL of each PCR reaction and incubated at 37 °C for 10 min. The PCR products were verified by an automated gel electrophoresis system, according to the AL320 method (QIAxcel DNA Screening Kit v2.0, QIAGEN, Hilden, Germany). In order to validate the results, a second PCR spanning another restriction site was performed using designed forward (891F_COI) and reverse (c1303R_COI) primers (Table S2). After the amplification, the digestion step was performed by adding EcoRV (New England Biolabs, Frankfurt, Germany), according to the manufacturer’s instructions.

#### 2.4.2. Characterization of Tpi Haplotypes Using DNA Sequencing

Plant host strain identification was additionally performed using the triosephosphate isomerase (*Tpi*) gene as a genetic marker, according to Nagoshi et al. (2019) [18]. The PCR amplification was performed according to Section 2.3, using the forward (TpiE4) and reverse (850R) primers described in Table S2. The PCR products were verified by an automated gel electrophoresis system, according to the OM500 method (QIAxcel DNA Screening Kit v2.0, QIAGEN), purified using PCR Clean-up Gel Extraction kit (Macherey-Nagel, Düren, Germany) and Sanger-sequenced by Eurofins Genomics (Cologne, Germany). The obtained *S. frugiperda Tpi* nucleotide sequences were aligned with the *Tpi* sequences for corn and rice variants according to the reference genome [13] (https://bipaa.genouest.org/data/public/sfrudb/), using Geneious software v. 10.2.3 (Biomatters Ltd., Auckland, New Zealand).

### 2.5. Target-Site Resistance Diagnostics by Pyrosequencing

Amino acid substitutions in the VGSC (T929I, L932F and L1014F), AChE (A201S, G227A and F290V), RyR (I4790M and G4946E) and ABCC2 (GC insertion and GY deletion) result in resistance to pyrethroid, carbamate/organophosphate, diamide and Cry1F Bt protein, respectively. Mutation sites in the VGSC, AChE and RyR are numbered according to *Musca domestica* (GenBank X96668), *Torpedo californica* (PDB ID: 1EA5) and *Plutella xylostella* (GenBank AET09964), respectively.

A pyrosequencing based genotyping assay was designed for targeting each mutation separately and performed across 34 FAW populations (see Table S1 for details about FAW populations).

Primer pairs were designed with Assay Design Software (QIAGEN, Hilden, Germany), according to sequences deposited at the National Center for Biotechnology Information (NCBI) for FAW para-type *VGSC* (GenBank KC435025) and *ace-1* (GenBank KC435023). Primers targeting FAW *RyR* (GenBank MK226188) and *ABCC2* (GenBank KY489760) were described elsewhere [28,30], as indicated in Table S2.

The PCR conditions for pyrosequencing were performed as described in Section 2.3, using primers given in Table S2. The pyrosequencing reaction was carried out as described elsewhere [33], using a sequencing primer specific for every target-site mutation analyzed, according to Table S2.

### 2.6. Fluorescence Based Allelic Discrimination Assays

#### 2.6.1. F290V Mutation in AChE

Primers were designed using the OligoArchitect™ Assay Design (Sigma-Aldrich, St. Louis, MO, USA) for the detection of the F290V mutation in ace1. Allele-specific probes were labeled with FAM (Sf_F290_FAM) or HEX (Sf_F290_mut_HEX) at the 5′ end for the detection of the wildtype and mutant allele, respectively (Table S2). Five individuals from populations from Brazil (Sf_Bra, MT-PL1 and PR-PG), Puerto Rico (PR60, PR61, PR62 and PR63), Kenya (EP-K, KV-K, MJ-K, KF-K and NW-K) and Indonesia (WS-I, DS-I, S-I, WC-I and JL-I) were tested (Table S1). PCR reactions and allele discrimination analysis were performed as described in Section 2.3.

#### 2.6.2. GC Insertion in ABCC2

The GC insertion in ABCC2 was detected according to Banerjee et al. (2017) [34], with slight modifications. Briefly, reactions were composed of a HEX-labeled probe (SfABCC2mut allele) that is *SfABCC2* mutant allele-specific and a FAM-labeled probe (SfABCC2), specific to the *SfABCC2* wildtype allele, gDNA (around 50 ng), the forward (Sf_ABCC2_F) and the reverse (Sf_ABCC2_R) primers (Table S2). The populations tested were the same as described in Section 2.6.1 and PCR reactions were prepared as mentioned in Section 2.3.

#### 2.6.3. I4790M Mutation in the RyR

The detection of the RyR I4790M mutation was performed as described by Boaventura et al. (2020) [30] using forward (Sf_taq_I4790_F) and reverse (Sf_taq_I4790_R) primers, the mutant allele-specific FAM-labeled probe (Sf_I4790_mut_FAM) and a HEX-labeled probe (Sf_I4790_HEX) that is wildtype allele-specific (Table S2). Individuals with known genotype from strain Chlorant-R (homozygote for M4790) as well as artificial heterozygotes (a mixture of gDNA from Chlorant-R and Sf_Bra individuals) were used as internal controls. The assay was validated with populations collected in Brazil, Puerto Rico, Kenya and Indonesia, as described above (Section 2.6.1).

## 3. Results

### 3.1. Characterization of S. frugiperda Strains

**Figure 2:**
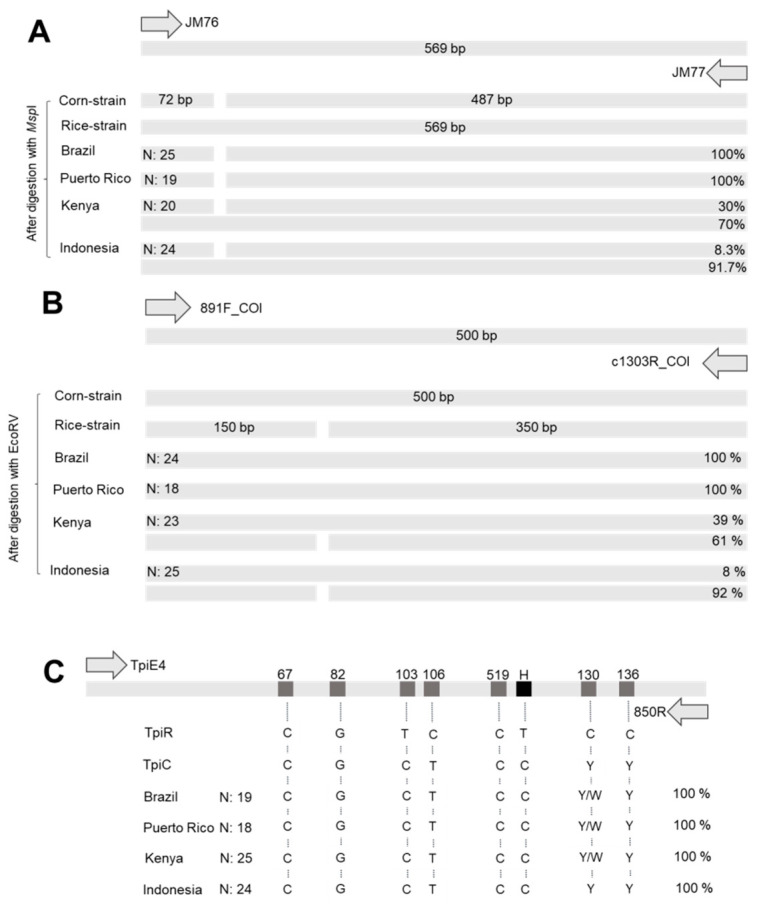
Schematic representation of amplified *COI* and *TpiE4* fragments used for *Spodoptera frugiperda* host plant strain identification in field samples collected in Brazil, Puerto Rico, Kenya and Indonesia. *COI* polymorphism in *S. frugiperda* was determined by RFLP-PCR. (**A**) PCR product containing a MspI restriction site in the corn strain and PCR fragments obtained after digestion with FastDigest MspI. (**B**) PCR product that contains an EcoRV strain-specific site. After digestion with EcoRV, the corn strain remains uncut, whereas the rice strain is cut. (**C**) *TpiE4* fragment with different polymorphic sites was Sanger-sequenced. Position marked H defines whether it is a rice strain (thymine; TpiR) or a corn strain (cytosine; TpiC).

The mitochondrial COI and nuclear Tpi molecular markers were employed for the identification of sympatric FAW rice and corn strain according to Nagoshi et al. [17,31,32]. The amplification of the respective COI fragment resulted in a PCR product of around 569 bp for both strains, but the fragment amplified from corn strain contained a MspI restriction site; therefore, after digestion, the PCR product was cut into two fragments (approximately 487 and 72 bp) (Figure 2A and Figure S1A). According to this method, all samples tested from Brazil and Puerto Rico were characterized as corn strain, whereas in Kenya and Indonesia, most of the individuals were characterized as rice strain, i.e., 70% and 91.7%, respectively (Figure 2A).

On the other hand, when using the EcoRV restriction site, the rice strain fragment was cut into two bands of around 350 and 150 bp and the corn strain fragment of 500 bp remained uncut (Figure 2B and Figure S1B). Again, all samples tested from Brazil and Puerto Rico were corn strain and most of the samples from Kenya and Indonesia were rice strain, i.e., 61 and 92%, respectively.

Host plant strain characterization using the *Tpi* gene was performed according to Nagoshi et al. (2019) [17]. The genetic markers used in this method are all single nucleotide substitutions present in the *TpiE4* exon. When using the primers 412F and 850R (Table S2), most of the *TpiE4* exon is amplified, producing a fragment of about 199 bp. Trimmed sequences were deposited in NCBI (GenBank MT706015–MT706018). The strain is defined by the gTpi183Y site, where the corn strain has a cytosine and the rice strain a thymine. All the samples tested from the four countries were corn strain, having a cytosine at the position gTpi183Y. It is worth mentioning that, at the position gTpi192Y, an adenine or thymine was observed in some samples from Brazil, Puerto Rico and Kenya (1, 1 and 2, respectively), while Nagoshi et al. (2019) [17] reported only a cytosine or a thymine at position Tpi192Y.

### 3.2. Detection of Target-Site Mutations by Pyrosequencing

The pyrosequencing assay used to genotype the mutations in the VGSC revealed that almost all analyzed larvae (n = 396) were wildtype, with no mutations at those sites analyzed. Only strain NB-KA from Indonesia included a few individuals heterozygous for the L1014F mutation, corresponding to 1.8% of all samples analyzed from Indonesia (Table 1). On the other hand, the mutations T929I and L932F were not detected at all in any population tested (Table 1), suggesting the lack of target-site resistance to pyrethroids in almost all samples analyzed. Resistant AChE alleles were found at much higher frequencies across countries in many populations analyzed. The mutation F290V was detected at the highest frequency (Table 1). In Brazil, 45% of the samples genotyped (57 out of 127 larvae) were heterozygote, whereas most samples from Puerto Rico (except strain PR65) were homozygote for V290, representing 85.7% of all samples tested (60 out of 70 larvae). Populations collected in Kenya and Indonesia also carried the F290V mutation in AChE and, on average, 47% and 56% of the samples were heterozygotes, respectively. The other AChE mutation sites analyzed, A201S and G227A, were not detected in Puerto Rico, while G227A was absent also in Kenya. RyR mutations G4946E and I4790M, conferring resistance to diamide insecticides, were not detected in any of the populations tested (Table 1); all individuals tested were homozygous wildtype at both positions. We also tested 379 individuals for the presence of a GY deletion in ABCC2, known to confer resistance to Cry1F in FAW [28]. This functionally validated target-site mutation was absent in samples collected in Puerto Rico, Kenya and Indonesia but detected in many of the tested Brazilian larvae, as recently described [28] (Table 1).

### 3.3. Detection of Target-Site Mutations by Fluorescence Based Allelic Discrimination Assays

**Figure 3:**
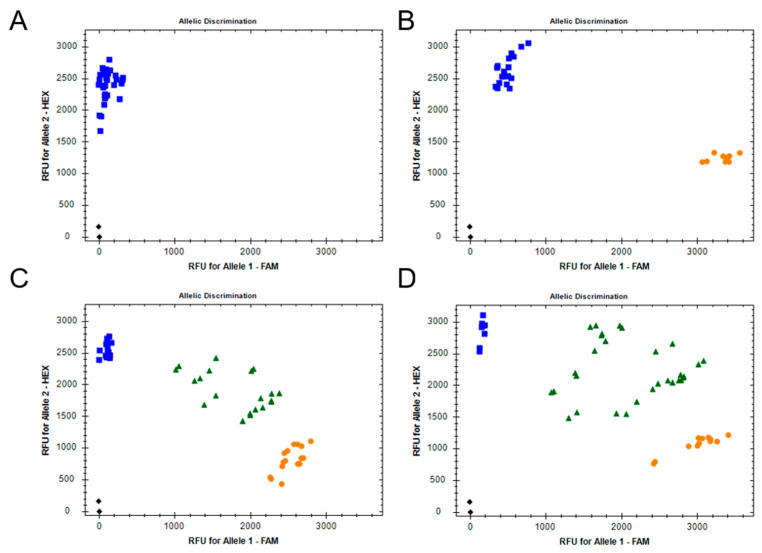
Bivariate plot showing the discrimination of different acetylcholinesterase alleles in *Spodoptera frugiperda* samples by an allele-specific real-time PCR fluorescent probe assay. Each dot represents a single larva. Blue squares represent mutant RR homozygotes (V290; allele 1), orange circles susceptible SS homozygotes (F290; allele 2) and green triangles SR heterozygotes (F290/V290). Analysis of fall armyworm field samples collected in (**A**) Puerto Rico, (**B**) Brazil, (**C**) Kenya and (**D**) Indonesia.

As the target-site, mutation F290V in AChE was the most frequent mutation found in all populations tested. We decided to develop a PCR-based allelic discrimination assay using fluorescent probes, which could be performed at larger-scale worldwide using a qPCR machine, because pyrosequencing-based diagnosis is more expensive and less common. All larvae analyzed from Puerto Rico were homozygote for the V290 resistance allele (Figure 3A). In Kenya and Indonesia, five populations were tested and, on average, 40% and 56% of the larvae were heterozygotes, respectively (Figure 3C,D). Individuals from the two field populations from Brazil (MT-PL1 and PR-PG) were homozygous for the V290 resistance allele (Figure 3B). The population Sf_Bra was kept for 15 years under laboratory conditions without insecticide exposure and all larvae were homozygotes for the susceptible wildtype allele F290 (Figure 3B).

**Figure 4:**
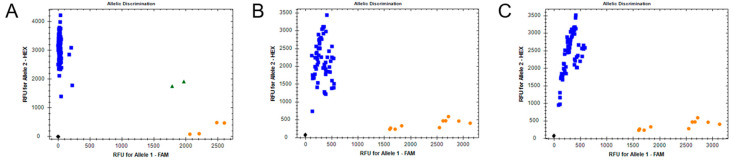
Detection of the RyR I4790M mutation using an allele-specific real-time PCR fluorescent probe assay, as recently described by Boaventura et al. (2020). (**A**) Genotyping of *Spodoptera frugiperda* collected in Puerto Rico represented by blue squares (wildtype SS homozygotes, I4790 allele), orange circles represent strain Chlorant-R mutant RR homozygotes from Brazil (M4790; allele 1) and green triangles artificial SR heterozygotes (I4790/M4790). All individuals tested from (**B**) Kenya and (**C**) Indonesia were susceptible homozygotes for I4790 (blue squares).

The I4790M mutation in the RyR was assessed using Chlorant-R resistant FAW larvae as a positive control for M4790. The resistant allele was not present in any other sample analyzed (Figure 4). For the detection of GC insertion at the ABCC2 causing resistance to Cry1F protein in *S. frugiperda* in Puerto Rico, the assay described by Banerjee et al. (2017) [34] was used, however substituting the VIC fluorescent probe with FAM, as described in Section 2.6.2. The GC insertion was only observed in Puerto Rican samples (Figure S2).

## 4. Discussion

The highly invasive nature and the potential economic impact of FAW have raised a lot of concerns across continents. Changes in agricultural practices and biological control are among a diverse range of measures implemented in recently invaded African countries and India by smallholder farmers and at rather low FAW infestation levels [25,26,35,36,37,38,39]. In countries with significant agricultural input subsidy programs, synthetic insecticides have been used to control FAW outbreaks [25,26,38]. However, in some countries, farmers claimed rather low efficacy of some of the insecticide classes used, such as organophosphates and pyrethroids [25,35]. It remains unclear whether the low field efficacy of insecticides against FAW in Africa is due to resistance or poor application technology affecting plant coverage.

Our genotyping study was conducted to shed some light on the presence of target-site insecticide resistance mechanisms in 34 populations collected in Kenya, Indonesia, Puerto Rico and Brazil. Our results from FAW populations collected in Kenya showed a relatively high frequency of the F290V mutation in AChE, the target of organophosphate and carbamate insecticides. The chance that alleles conferring resistance to these rather old chemical classes were already present at high frequency in the invasive population is quite high. Their frequency was likely augmented by further selection, using applications of cheap products based on organophosphate and carbamate chemistries. Similar findings have been reported for the tomato leafminer (*Tuta absoluta*) in Iran, where this pest has been recently introduced. Resistance to pyrethroids and organophosphates was expected in the invading populations and this expectation was supported by the identification of target-site mutations in VGSC and ace1, respectively [40].

As a result of frequent insecticide applications, multiple resistance cases have been described for field populations in regions where FAW is native [41,42]. In Brazil and Puerto Rico, for instance, resistance has been reported to pyrethroids, organophosphates, carbamates, spinosyns, benzoylureas and, most recently, diamides [29,42,43,44,45,46,47]. The genetic inheritance of insecticide resistance in FAW has been investigated, and cases of FAW resistance were described as polygenic and metabolic [46,47,48].

Pyrethroid insecticides are supposed to bind in the domain IIS4-S5 linker and domain IIIS6 of *para*-type sodium channels [49] and the common L1014F mutation has been reported to confer pyrethroid resistance ratios of 10-20-fold [29,50,51,52]. More than 30 unique resistance-associated mutations including L1014F or combinations thereof have been described in VGSC in many other different species [53]. Three mutations (T929I, L932F and L1014F) at the VGSC have been recently described in pyrethroid-resistant *S. frugiperda* from Brazil [29] and one of the mutations, L932F, was detected in FAW populations from China [54]. Our genotyping results revealed the absence of the L1014F mutation in almost all analyzed samples, except for one population from Indonesia (K-I, Table S1), where only two heterozygotes out of 30 individuals were detected. No other mutation conferring pyrethroid resistance and described for *S. frugiperda* was detected in the populations tested. However, other mechanisms such as enhanced metabolism by elevated levels of cytochrome P450 monooxygenases are known to confer pyrethroid resistance in FAW [29] but were not tested in our study as we used gDNA of alcohol preserved FAW samples as we did not have access to living insects.

Organophosphates and carbamates target AChE and resistance is often associated with mutations in the *ace-1* gene, leading to amino acid substitutions at the enzyme’s active site [55]. Our genotyping results confirmed the presence of the following amino acid substitutions A201S, G227A and F290V in populations collected in Brazil, as described by Carvalho et al. (2013) [29]. The detected point mutations co-exist, at least in heterozygous individuals, in populations BA-SD, PR-PG and MT-PL1-2. Moreover, we also detected the F290V mutation in samples from Puerto Rico, Indonesia and Kenya. Point mutations linked to organophosphate resistance have been described for *Cydia pomonella* (F399V), *Chilo suppressalis* (A314S) and *P. xylostella* (D131G, A201S, G227A and A441G) [56,57,58,59,60]. Moreover, heterologous expression of AChE mutants (A303S, G329A and L554S) from the silkworm (*Bombyx mori*) have supported the reduction in AChE sensitivity towards carbamate and organophosphate insecticides [61].

Diamide insecticides comprised two chemotypes, the phthalic (flubendiamide) and anthranilic acid diamides (e.g., chlorantraniliprole), which were shown to be affected differently by the presence of point mutations leading to amino acid substitutions, particularly G4946E and I4790M in the lepidopteran RyR (numbering according to the *P. xylostella* RyR)—recently reviewed by Richardson et al. [62]. Phthalic diamides are less potent against pests carrying a methionine at position 4790 [63]. In Puerto Rico, resistance ratios of 160 to 500- fold have been reported to chlorantraniliprole and flubendiamide, respectively, but the mechanisms of resistance were not studied in detail [42]. However, in our genotyping assays, G4946E and I4790M were not detected in samples from Puerto Rico or any other country. So far, the I4790M mutation in *S. frugiperda* has been detected only in one FAW strain (Chlorant-R) from Brazil, selected with chlorantraniliprole under laboratory conditions and showing resistance ratios of >230 and >42,000-fold against chlorantraniliprole and flubendiamide, respectively [30,43]. Our genotyping data suggest that the frequency of those resistance alleles (G4946E and I4790M) is low under field conditions in those locations here investigated.

Mutations in the ABCC2 transporter have been associated with Cry1F and Cry1A.105 resistance in FAW: a GC insertion causing a premature stop codon has been found in Cry1F-resistant FAW strains from Puerto Rico [34,64], whereas a functionally validated GY deletion was very recently described in Cry1F-resistant populations from Brazil [28]. While we identified the described GC insertion and GY deletion in many samples from Puerto Rico and Brazil, respectively, none of the above-mentioned mutations were found in populations from Kenya or Indonesia, supporting the absence/very low frequency of these mutations in the field and a lack of selection pressure by transgenic corn expressing Cry1F in those countries. Recent whole-genome sequencing of FAW samples collected in China, Malawi, Uganda and Brazil revealed a novel ABCC2 resistance allele in FAW collected in Brazil, leading to a truncated and likely non-functional protein [65].

Two sympatric host plant strains of *S. frugiperda* have been previously described: the corn strain and the rice strain, which prefers forage grasses and rice [9,10,11]. Recent studies have reported that *S. frugiperda* populations present in Asia and Africa are an inter-strain hybrid, with the genetic background mostly from the corn strain [54,66]. Therefore, we were interested in the host-plant strain composition of our samples and analyzed individual larvae of different populations using recently described markers by RFLP and PCR. Our results using COI and Tpi genetic markers confirmed that the corn strain is the most abundant in Brazil and Puerto Rico, as shown in previous studies [14,15,67,68]. The COI genetic marker used in this study revealed dominance of the rice strain in those populations that we collected in Kenya (70%, though collected from corn plants) and Indonesia (91.7%). However, to avoid any identification bias, we used a second marker, *Tpi*, and the obtained data revealed that all samples, including those from Kenya and Indonesia, resemble corn strain and none rice strain. This discrepancy between COI and *Tpi* markers has already been noticed by other authors, especially with samples from Africa and Asia, where strain characterization is dependent on the molecular marker used [16,18,65]. However, the exclusive identification of the corn strain in our samples by using the *Tpi* marker is in accordance with the preferred host, suggesting that it is a more accurate strain marker than COI. In terms of insecticide susceptibility, there is not much difference between host-plant strains, at least when considering the efficacy of recommended label rates of many insecticides. A slightly higher level of cytochrome P450 activity in corn-adopted FAW [9] may render the corn strain slightly more tolerant, but this is unlikely to result in reduced efficacy of insecticides at their recommended label rates in the absence of resistance. The rice strain has been reported to be more susceptible to diazinon, carbaryl and Bt toxins, whereas corn strain larvae were shown to be more susceptible to the carbamate carbofuran [12,19,20]. Recently, Arias et al. (2019) [14] have tested the possible influence caused by the migration of individuals from hot spots—characterized by higher LC_50_ values against flubendiamide and lufenuron. The authors concluded that migration did not play the key role but, rather, the pest management measures adopted and cropping strategies in the respective region. Therefore, we want to reinforce that, although high frequencies of alleles conferring resistance to organophosphate and carbamates were detected, the choice of the appropriate management strategy to be adopted based on regionally registered insecticides and alternative measures is likely to be the key factor for sustainable FAW control. The practical relevance of the presence of alleles conferring resistance is determined by the selection pressure adopted in the field and whether the mutations present carry any fitness cost. The resistance alleles might decrease in frequency in the absence of selection pressure or increase when the application of specific insecticides increases [14]. Therefore, strategies to slow down the development of insecticide resistance should be driven by the application of insecticides with different modes of action [14,69]. Compounds such as diamides, emamectin benzoate and spinosyns [25,70,71,72] have mostly shown good control of several lepidopteran pests and would be valuable tools in FAW resistance management strategies in the newly invaded countries.

## 5. Conclusions

Based on our genotyping results described in this study, the field efficacy of organophosphate and carbamate insecticides is likely to be compromised by the presence of the AChE V290 allele in hetero- and homozygous form in Brazil, Kenya, Indonesia and Puerto Rico. To achieve successful integrated pest management of FAW and reduce the risk of economic losses, resistance management strategies will need to be implemented at regional levels in the newly invaded countries and can be supported by using the presented diagnostic tools to detect and monitor the early presence of resistance alleles in the field.

## Figures and Tables

**Table 1:** Genotyping by pyrosequencing for different target-site mutations in major insecticide targets. In total, larvae of 34 populations from Brazil, Puerto Rico, Kenya and Indonesia were analyzed. Homozygous susceptible (SS), heterozygotes (RS) and homozygous resistant (RR).

Target	Country	Mutation	N	SS (%)	RS (%)	RR (%)
Voltage-gated sodium channel (VGSC)	Brazil	L1014F	140	100.0	0.0	0.0
	Puerto Rico		70	100.0	0.0	0.0
	Kenya		76	100.0	0.0	0.0
	Indonesia		110	98.2	1.8	0.0
	Brazil	L932F	143	100.0	0.0	0.0
	Puerto Rico		64	100.0	0.0	0.0
	Kenya		75	100.0	0.0	0.0
	Indonesia		88	100.0	0.0	0.0
	Brazil	T929I	143	100.0	0.0	0.0
	Puerto Rico		64	100.0	0.0	0.0
	Kenya		75	100.0	0.0	0.0
	Indonesia		88	100.0	0.0	0.0
Acetylcholinesterase (AChE)	Brazil	A201S	147	92.5	4.1	3.4
	Puerto Rico		29	100.0	0.0	0.0
	Kenya		76	89.5	10.5	0.0
	Indonesia		85	77.6	22.4	0.0
	Brazil	F290V	127	55.1	44.9	0.0
	Puerto Rico		70	4.3	10.0	85.7
	Kenya		76	26.3	47.4	26.3
	Indonesia		86	19.8	55.8	24.4
	Brazil	G227A	161	55.3	32.3	12.4
	Puerto Rico		29	100.0	0.0	0.0
	Kenya		76	100.0	0.0	0.0
	Indonesia		86	83.7	16.3	0.0
Ryanodine receptor (RyR)	Brazil	G4946E	140	100.0	0.0	0.0
	Puerto Rico		70	100.0	0.0	0.0
	Kenya		76	100.0	0.0	0.0
	Indonesia		90	100.0	0.0	0.0
	Brazil ^a^	I4790M	140	100.0	0.0	0.0
	Puerto Rico		70	100.0	0.0	0.0
	Kenya		76	100.0	0.0	0.0
	Indonesia		90	100.0	0.0	0.0
ATP-binding cassette transporter subfamily C (ABCC2)	Brazil ^b^	GY del	211	39.83	14.30	45.87
	Puerto Rico		19	100.0	0.0	0.0
	Kenya		70	100.0	0.0	0.0
	Indonesia		79	100.0	0.0	0.0

^a^ Data published by Boaventura et al. (2020a) [30]; ^b^ Data published by Boaventura et al. (2020b) [28].

## Supplementary Materials

The following are available online at https://www.mdpi.com/2075-4450/11/8/545/s1, Table S1: Populations of *Spodoptera frugiperda* collected in different countries and years used for genotyping of target-site mutations, Table S2: List of primers for pyrosequencing and dual fluorescence probe assay used for the identification of different target-site mutations and Spodoptera frugiperda strain identification by RFLP-PCR and Sanger sequencing, Figure S1: Automated analysis of DNA fragments showing COI polymorphism in Spodoptera frugiperda. (A) PCR product containing a strain specific Mspl site that was amplified using the JM76 and JM77 primers (Table S2) followed by products obtained after the digestion with FastDigest MspI. Corn-strain is cut and rice-strain remains uncut as it does not have the Mspl site. (B) PCR product amplified with the primers 891F_COI and c1303R_COI (Table S2) that contains a EcoRV strain specific site. After digestion with EcoRV the corn-strain amplicon remains uncut whereas it is cut in the rice-strain. Details about samples, see Table S1, Figure S2: Detection of GC insertion allele at the ATP-binding cassette subfamily C2 (ABCC2) conferring resistance to Bacillus thuringiensis Cry1F toxin using PCR fluorescent probe assay described by Banerjee et al [34]; Blue squares represent mutant ABCC2 homozygotes for the GC insertion, orange circles ABCC2 wildtype SS homozygotes, and green triangles SR representing heterozygotes. Analysis of fall armyworm field samples collected in (A) Brazil, (B) Puerto Rico, (C) Kenya, and (D) Indonesia.
