# Supplementary material for: Monitoring of Target-Site Mutations Conferring Insecticide Resistance in Spodoptera frugiperda
Source: Insects. 2020 Aug 18;11(8):545. doi: 10.3390/insects11080545 (PMC7469220; doi:10.3390/insects11080545)
Supplement: Supplementary file 1 [file insects-11-00545-s001.pdf]

Table S1. Populations of *Spodoptera frugiperda* collected in different countries and years used for genotyping of target-site mutations.

Country	Sample ID	City. State	Year	Host plant
Brazil[1]	Sf_Bra	Unknown, São Paulo	2005	corn
	Sf_Cor	Correntina, Bahia	2016	corn
	Sf_Des	São Desidério, Bahia	2016	corn
	PR-PG	Ponta Grossa, Paraná	2018	corn
	SP-IT	Ituverava, São Paulo	2018	corn
	MS-CS	Chapadão do Sul, Mato Grosso do Sul	2018	corn
	MT-SZ	Sapezal, Mato Grosso	2018	corn
	MT-TS	Tangará da Serra, Mato Grosso	2018	corn
	MT-PL1-2	Primavera do Leste, Mato Grosso	2018	corn
	MT-LV	Lucas do Rio Verde, Mato Grosso	2018	corn
	BA-SD	São Desidério, Bahia	2018	corn
	RO-VI	Vilhena, Rondônia	2018	corn
Indonesia	WS-I	Padang Pariaman, Sumatra	2019	corn
	DS-I	Deli Serdang, Sumatra	2019	corn
	S-I	Simalungun, Sumatra	2019	corn
	WC-I	Waled Cirebon, Java	2019	corn
	BC-I	Babakan Cirebon, Java	2019	corn
	JL-I	Jati Agung, Lampung	2019	corn
	SB-I	Saputih Banyak, Lampung	2019	corn
	K-I	Kediri, Java	2019	corn
	B-I	Blitar, Java	2019	corn
Kenya	EP-K	Eldoret	2019	corn
	KV-K	Kisii	2019	corn
	NJ-K	Nakuru	2019	corn
	MJ-K	Muranga	2019	corn
	MD-K	Mombasa	2019	corn
	KF-K	Kajiado	2019	corn
	BA-K	Bungoma	2019	corn
	NW-K	Narok	2019	corn
Puerto Rico	PR60	Ponce	2017	corn
	PR61	Ponce	2017	corn
	PR62	Ponce	2017	corn
	PR63	Ponce	2017	corn
	PR64	Ponce	2017	corn

[1] Samples were described in Boaventura et al. (2020) [1] and Boaventura et al. (2020) [2].

Table S2. List of primers for pyrosequencing and dual fluorescence probe assay used for the identification of different target-site mutations and Spodoptera frugiperda strain identification by RFLP-PCR and Sanger sequencing.

Target	Mutation	Primers	Sequence (5'- 3')	Annealing Temperature (°C)	Assay
Ryanodine receptor	G4946E[1]	Sf_G4946_F	GTGATGGGCAACTTCAAC	50	Pyrosequencing
		Sf_G4946_R.btn	[btn]TTTTCCGTTATGCGTGAC		
		Sf_G4946_F.Seq	ATTTGCTAGATGTCGCT		
	I4790M[1]	Sf_I4790_F.btn	[btn]CGAGGACTTCTTCTACATGG-	50	
		Sf_taq_I4790_R	CACCTTGAGATGATAGTACC		
		Sf_I4790_R.Seq	ATGGTAGTACCCGATGA		
	I4790M[1]	Sf_taq_I4790_F	ACGACGATGCACTAGAAG	60.6	Probe assay
		Sf_taq_I4790_R	CACCTTGAGATGATAGTACC		
		Sf_I4790_HEX	[HEX]TGTCGCTCGCTATACTCATCG[BHQ1]		
		Sf_I4790_mut_FAM	[6FAM]CTCGCTATGCTCATCGGGT[BHQ1]		
Voltage-gated sodium channel	L1014F	Sf_L1014_F	TCTTCCTGGCTACAGTCG	50	Pyrosequencing
		Sf_L1014_R.btn	[btn]GACAGTAACAGGGCCAAG		
		Sf_L1014_Seq	CAGTCGTCATYGGCA		
	L932F/T929I	Sf_L932_T929_F.btn	[btn]TAATGGGTAGGACAATGG	53	
		Sf_L932_T929_R	AATCCACGTAATTTTTCC		
		Sf_L932_T929_R.Seq	AAATATGAAAATAATGATGC		
Acetylcholinesterase	F290V	Sf_F290_F	GCATCCGATTAGCAGAAG	52	Pyrosequencing
		Sf_F290_R	[btn]TATGATGGGCACAAAAGG		
		Sf_L932_F	GAACTCTTGGTATTTGTGA		
		Sf_taq_F290_F	CCAGATGAACTAGTCAATAATG	60	Probe assay
		Sf_taq_F290_R	GGAACGAACCATCTATGA		
		Sf_F290_FAM	[FAM]TATTTGTGAATTTCCTTTTGTGCCC[BHQ1]		
		Sf_F290_mut_HEX	[6HEX]TATTTGTGAAGTTCCTTTTGTGCCC[BHQ1]		
	A201S / G227A	Sf_A201S_ G227A_F	TTTGATACCCCTGATGTACC	53	Pyrosequencing
		Sf_A201S_ G227A_R	[btn]AATGAAACCGAAACTGCTC		
		Sf_A201S_Seq	TAACATTATTCGGTGAGTC		
		Sf_G227A _Seq	GGCGATAATGCAGTCA		
ATP-binding cassette transporter subfamily C2	GY deletion[2]	Sf_788-Gydel_F	[btn] CCGACTACTGGCTTAGTTT	50	Pyrosequencing
		Sf_788-Gydel_R	GCTCGCATAGTCATCACT		
		Sf_788-GYdel_seq	CTTCGGGTAAAGTTTGT		
	GC insertion[3]	Sf_ABCC2_F	TGGAGGCCGAAGAGAGACA	50	Probe assay
		Sf_ABCC2_R	AGGAGTTGACTGACTTCATGTACCT		
		SfABCC2mut allele	[HEX]AAGCACATCGCCCACTT[BHQ1]		
		SfABCC2	[6FAM]CCAAGCACATCCCACTT [BHQ1]		
Mitochondrial cytochrome oxidase subunit I		JM76[5]	GAGCTGAATTAGGRACTCCAGG	60	PCR-RFLP

	JM77[5]	ATCACCTCCWCCTGCAGGATC		
	891F_COI[5]	TACACGAGCATATTTTACATC	52	
	c1303R_COI[5]	CAGGATAGTCAGAATATCGACG		
Triosephosphate isomerase	TpiE4[6]	CCGGACTGAAGGTTATCGCTTG	56	PCR-Seq
	850R[6]	AATTTTATTACCTGCTGTGG		

Primers described by [1] Boaventura et al. (2020) [2];[2] Boaventura et al. (2020) [1]; [4] Banerjee et al. (2017) [3]; [5] Nagoshi et al. (2017) [4]; [6] Nagoshi et al. (2019) [5].

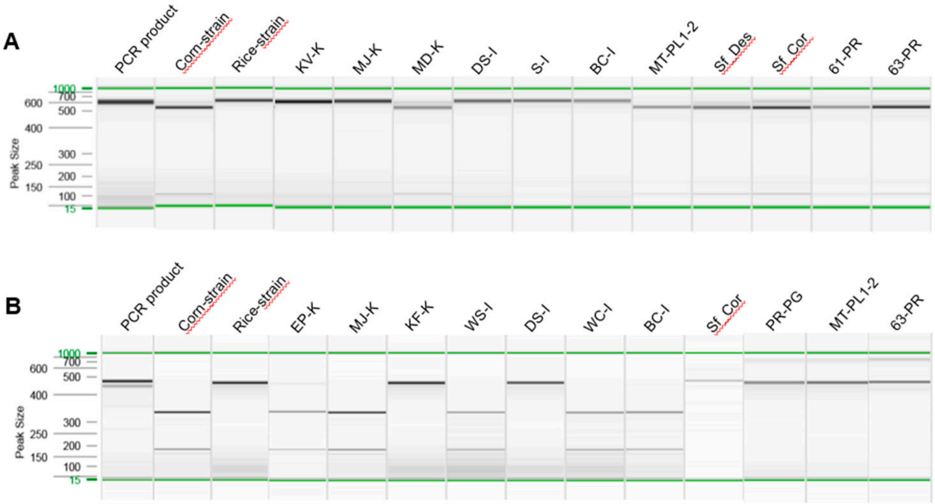

Figure S1. Automated analysis of DNA fragments showing *COI* polymorphism in *Spodoptera frugiperda*. (**A**) PCR product containing a strain specific *Mspl* site that was amplified using the JM76 and JM77 primers (Table S2) followed by products obtained after the digestion with FastDigest *MspI*. Corn-strain is cut and rice-strain remains uncut as it does not have the *Mspl* site. (**B**) PCR product amplified with the primers 891F_COI and c1303R_COI (Table S2) that contains a EcoRV strain specific site. After digestion with EcoRV the corn-strain amplicon remains uncut whereas it is cut in the rice-strain. Details about samples, see Table S1.

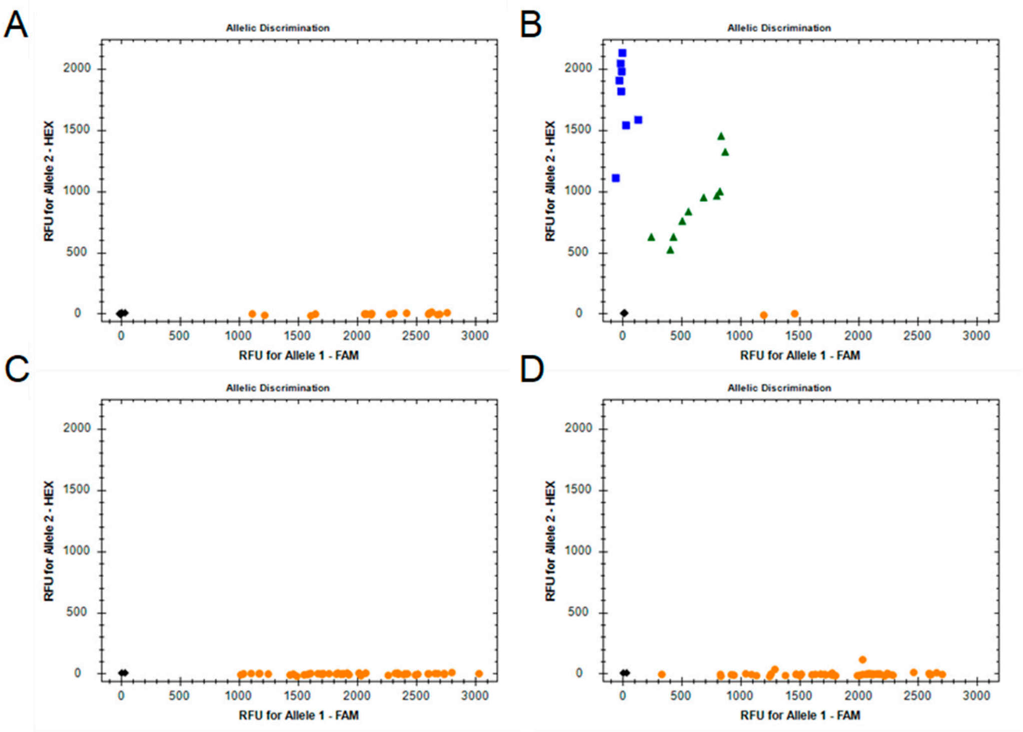

Figure S2. Detection of GC insertion allele at the ATP-binding cassette subfamily C2 (ABCC2) conferring resistance to *Bacillus thuringiensis* Cry1F toxin using PCR fluorescent probe assay described by Banerjee et al [3]; Blue squares represent mutant ABCC2 homozygotes for the GC insertion, orange circles ABCC2 wildtype SS homozygotes, and green triangles SR representing heterozygotes. Analysis of fall armyworm field samples collected in (**A**) Brazil, (**B**) Puerto Rico, (**C**) Kenya, and (**D**) Indonesia.

References

1. Boaventura, D.; Ulrich, J.; Lueke, B.; Bolzan, A.; Okuma, D.; Gutbrod, O.; Geibel, S.; Zeng, Q.; Dourado, P.M.; Martinelli, S.; et al. Molecular characterization of Cry1F resistance in fall armyworm, *Spodoptera frugiperda* from Brazil. *Insect Biochem. Mol. Biol.* **2020**, *116*, 103280, doi:10.1016/j.ibmb.2019.103280.
2. Boaventura, D.; Bolzan, A.; Padovez, F.E.; Okuma, D.M.; Omoto, C.; Nauen, R. Detection of a ryanodine receptor target-site mutation in diamide insecticide resistant fall armyworm, *Spodoptera frugiperda*. *Pest. Manag. Sci.* **2020**, *76*, 47–54, doi:10.1002/ps.5505.
3. Banerjee, R.; Hasler, J.; Meagher, R.; Nagoshi, R.; Hietala, L.; Huang, F.; Narva, K.; Jurat-Fuentes, J.L. Mechanism and DNA-based detection of field-evolved resistance to transgenic Bt corn in fall armyworm (*Spodoptera frugiperda*). *Sci. Rep.* **2017**, 7, doi:10.1038/s41598-017-09866-y.
4. Nagoshi, R.N.; Koffi, D.; Agboka, K.; Tounou, K.A.; Banerjee, R.; Jurat-Fuentes, J.L.; Meagher, R.L. Comparative molecular analyses of invasive fall armyworm in Togo reveal strong similarities to populations from the eastern United States and the Greater Antilles. *PLoS ONE* **2017,** 12, e0181982, doi:10.1371/journal.pone.0181982.
5. Nagoshi, R.N.; Goergen, G.; Plessis, H.D.; Van den Berg, J.; Meagher, R. Genetic comparisons of fall armyworm populations from 11 countries spanning sub-Saharan Africa provide insights into strain composition and migratory behaviors. *Sci. Rep.* **2019**, *9*, 8311, doi:10.1038/s41598-019-44744-9.
